# Supplementary material for: The cost-effectiveness of a two-step blood pressure screening programme in a dental health-care setting
Source: PLoS One. 2021 May 25;16(5):e0252037. doi: 10.1371/journal.pone.0252037 (PMC8148372; doi:10.1371/journal.pone.0252037)
Supplement: Attachment — Submitted filename: Artikel cost-effectiveness 201001.docx [file pone.0252037.s002.docx]

RESEARCH ARTICLE

**The cost-effectiveness of a two-step blood pressure screening programme in a dental health-care setting**

**Helen Andersson^1,2^, Mikael Svensson ^2^ & Håkan Bergh^2,3^**

^1^Hallands Hospital Varberg, Sweden, ^2^School of Public Health and Community Medicine, Institute of Medicine, University of Gothenburg, Sweden, ^3^Dept. of Research & Development Unit, Hospital Varberg, Region Halland, Sweden.

**Abstract**

**Background**

Hypertension is one of the largest contributors to the disease burden and a major economic challenge for health-care systems. Early detection of persons with high blood pressure can be achieved through screening and has the potential to reduce morbidity and mortality. We evaluate the cost-effectiveness of an opportunistic hypertension screening programme in a dental-care facility for individuals aged 40-75 relative to the no-screening baseline scenario.

**Methods**

A cost-effectiveness analysis (CEA) was carried out from the payer and societal perspectives, and the short-term cost per identified case of hypertension and long-term cost per quality-adjusted life year (QALY) were reported. Data on the short-term cost were based on a real-world screening programme in which 2025 healthy individuals were screened for hypertension. Data on the long-term cost were based on the short-term outcomes combined with modelling in a Markov cohort model. Deterministic and probabilistic sensitivity analyses were carried out to assess uncertainty.

**Results**

The short-term analysis showed an additional cost of 4,800 (12,800) SEK per identified case of hypertension from the payer (societal) perspective (€470 and €1,240). The long-term analysis showed a payer (societal) cost per QALY of 2.2 million (2.8 million) SEK (€210,000 and €270,000).

**Conclusion**

The long-term model results showed that the screening model is unlikely to be cost-effective in a country with a well-developed health-care system and a relatively low prevalence of hypertension.

**Keywords:** hypertension, blood pressure, screening, cost-effectiveness analysis

**Introduction**

Hypertension or high blood pressure (BP) is an important worldwide public health problem and the most important risk factor for the total disease burden worldwide [1], with its sequelae including stroke and myocardial infarction [2]. It is estimated that 10% of health-care spending is directly related to hypertension and its complications [3]. The overall prevalence of hypertension in adults is approximately 25-45% in Europe [4]. In Sweden, high BP affects an estimated 1.8 million people, representing 27% of the adult population [5]. Since there are effective treatments that reduce both high BP and an individual's risk of developing sequelae [6], it is important to identify those individuals who have high BP as early as possible. Early detection of individuals with high BP may be done through BP screening among "healthy" individuals.

One type of screening is opportunistic screening, whereby a patient utilizes a health-care facility for another reason and, in addition to the regular treatment related to the visit, receives BP screening. Since a majority of the population (80% in Sweden) regularly seeks dental-care services in the form of annual check-ups [7], dental-care service providers can be a possible provider of screening for hypertension, as shown in several studies [8-10].

There is a knowledge gap on the optimal population screening programme for detecting hypertension [2], and there is a great need to evaluate the long-term cost-effectiveness of such programmes. One such initiative, an opportunistic two-step screening of hypertension, was tested during the dental-care visits of a general population, resulting in a positive predicted value of 0.76 and a reduction of the false positive values by 86% via a second step of home BP measurement [10]. The cost-effectiveness of an opportunistic screening programme for high blood pressure in a general population has not previously been assessed. Thus, the aim of this study was to evaluate the cost-effectiveness of initiatives such as the aforementioned opportunistic two-step hypertension screening programme.

**Methods**

**The Intervention**

A two-step BP screening was conducted at four different dental clinics in a region of southern Sweden. The intervention was a single-arm screening programme implemented in an unscreened population, and the no-screening comparator group in this evaluation is assumed to be characterized by the status quo in which blood pressure tests are carried out when individuals visit health-care facilities. In the screening, BP was measured after five minutes of rest by a dental nurse twice in both arms (first step), and those with a mean BP value ≥140 and/or ≥90 mmHg continued with home BP measurement for one week (twice in the morning and in the evening) (second step). If the home BP resulted in a mean value ≥135 and/or ≥85, the individuals were referred to a primary health-care centre (PHC) for further assessment and diagnosis. Of the 2025 screening participants (exclusion: hypertension, diabetes, atrial fibrillation), 170 (8%) were newly identified with hypertension, resulting in a number needed to screen (NNS) of 12 and a positive predicted value (PPV) of 0.76. The second step with home BP measurement eliminated 86% of the false positives [10].

**Cost-effectiveness analysis**

The analysis evaluates the two-step screening programme compared to the no-screening baseline in terms of short- and long-term outcomes, where the short-term analysis uses an intermediate outcome measure, identified hypertension patients, and the long-term analysis uses quality-adjusted life years (QALYs) as the outcome metric.

The result is presented in terms of the ICER (incremental cost-effectiveness ratio), which is the difference in costs divided by the difference in health outcomes with the screening programme compared to no-screening baseline scenario:${{(Cost}_{SCREENING}-{Cost}_{NO SCREENING})}/{{(Outcome}_{SCREENING}-{Outcome}_{NO SCREENING})}.$

Sub-group analyses of the screening programme based on sex are carried out considering the sex differences in the incidence of hypertension-related conditions, especially acute myocardial infarction (AMI). Moreover, cost-effectiveness is evaluated from a societal as well as a payer perspective. The difference between the two perspectives is that the societal perspective also includes the costs of the programme for the included individuals (primarily time-use and travel-related costs). All costs are expressed in 2019 prices in Swedish kronor (SEK), and the main results are also presented in euro (EUR) assuming an exchange rate of 1 EUR = 10.3 SEK.

**Short-term analysis**

The short-term analysis includes the time frame up until persons are potentially diagnosed with hypertension and thus estimates the ICER in terms of the cost of identifying one patient with hypertension through the screening programme.

The model relies on the data from the primary screening study [10]. The formal dental and health-care cost data (Table 1) include the blood pressure test costs in dental and primary health-care facilities, ECG costs, and laboratory and diagnostic costs. Non-health-care costs include patient time costs and travel costs. Patient time costs refer to the time spent on the blood pressure tests in the dental-care setting and, for patients referred to the PHC, also the time spent in this latter setting. We assume that the visits did not displace working hours for the patients and thus value each hour of patient time based on average net wages [11]. Travel costs (to the PHC) are based on the average distance (3 km) and a cost of 1.85 SEK per km.

The number of newly discovered cases of hypertension in the screening group (170 individuals) is compared with a corresponding number in a hypothetical comparator arm (46 individuals). The parameter value in the comparator arm is based on an assumption that 61 (expected incidence 3%) [12, 13] of the 2025 individuals would have been identified as having high blood pressure during a visit to a primary care centre for some reason (on the patient's own initiative, at a doctor’s suggestion, or for other reasons). Among those diagnosed with high blood pressure based on blood pressure screening in a clinical setting, we estimate that approximately 15 individuals (25%) present false positives (that is, they display white coat hypertension [WCHT]), and the remaining 46 (75%) are expected to be true positives.

**Long-term analysis: Markov-cohort model**

For long-term costs and health outcomes, we developed a Markov-cohort model with the structure shown in Figure 1. At the time of the introduction of the screening programme, the entire cohort is in the “Healthy” state, which is also the status quo of the comparator case without the screening programme. Consequently, there are annual (one-year-cycle) risks of an AMI or stroke incident based on the risk equations from the Framingham studies adjusted for age, sex, lipid levels, and diastolic blood pressure [14]. There is also an annual age- and sex-adjusted risk of mortality from other causes (not AMI or stroke) based on Swedish life-table data. From the AMI and stroke health states, there is either death as a direct consequence of the event or a transition to the post-AMI or post-stroke state. The time perspective of the Markov model is 20 years, with annual discounting of costs and health outcomes of 3%, in line with the recommendations for cost-effectiveness analyses in Swedish health policy settings [15] (see Table 1 for input data on costs and transition probabilities). Health outcomes are measured in terms of quality-adjusted life years (QALYs), which combine health-related quality of life (QALY weights) and life length [16]. QALY weights are indexed such that 0 is interpreted as “equal to being dead” and 1 is interpreted as “the best possible health state”. Table 1 lists the QALY-weight decrements associated with a stroke and AMI event.

**Assessing uncertainty**

Parameter uncertainty analyses were carried out using (one-way) deterministic sensitivity analysis (DSA) and probabilistic sensitivity analysis (PSA) based on 5,000 Monte Carlo simulations. The results from the DSA are shown using a Tornado diagram where the ICER intervals are based on varying parameter input values for the time horizon of the Markov cohort model, underlying hypertension prevalence in the cohort, AMI and stroke costs, drug treatment costs, and QALY-weight decrements for AMI and stroke events.

The PSA assesses the uncertainty with jointly varying parameter values for hypertension prevalence, costs, transition probabilities, and QALY-weight decrements. The results from the PSA are shown using a cost-effectiveness acceptability curve (CEAC), which shows the probability that the screening programme (compared to the no-screening baseline) is cost-effective at different levels of the maximum willingness to pay per QALY (“threshold value”). All uncertainty ranges and distributions are listed in Table 1.

**Results**

Table 2 shows the short-term cost with and without the screening programme from a payer (health and dental care) as well as societal perspective. The increase in costs with the total screening programme is approximately 0.6 million SEK (€58,000) from a payer perspective and 1.6 million SEK (€154,000) from a societal perspective. The increase in costs in individual terms (total cost divided by the cohort size) with the screening programme is 295 SEK (€29) in a payer perspective and 785 SEK (€76) in a societal perspective.

As previously reported in the main publication on the screening programme [10], from the cohort of 2025 persons, the screening programme identified 170 persons as having true hypertension compared to an estimated 46 persons who would have been identified in the absence of the screening programme. The additional 124 persons correctly identified as having hypertension implies an incremental cost per identified hypertension case at approximately 4,800 SEK (payer perspective) and 12,800 SEK (societal perspective) (€470 and €1,240).

Table 3 shows the long-term costs and health outcomes in the full cohort as well as for men and women separately (assuming equal cohort size). The results show that the incremental cost with the screening programme is 3.9–4.9 million SEK (€380,000–€475,000). If we consider an all-male cohort, the incremental cost would be 4.4–5.3 million SEK (€430,000–€515,000), and in an all-female cohort, it would be 3–4 million SEK (€290,000–€390,000). The lower value in the range refers to the payer perspective, and the higher value refers to the societal perspective.

The QALY gain is estimated at 1.77 for the entire cohort but higher (3.18) if we assume an all-male cohort and lower (0.66) if we assume an all-female cohort. The better health outcomes for an all-male cohort are based on the higher hypertension prevalence as well as the higher (untreated) AMI risk among men.

The associated cost per gained QALY is approximately 2.2 million SEK (€210,000) in a payer perspective and 2.8 million SEK (€270,000) in a societal perspective. Considering an all-male cohort, the estimated results are 1.4 and 1.7 million SEK per QALY (€135,000 and €165,000). For an all-women cohort, the cost is estimated at 4.6 and 6.1 million SEK per QALY (€445,000 and €590,000).

**Deterministic sensitivity analysis**

Figure 2 shows the one-way deterministic sensitivity analysis when we vary the input parameter values for the model time horizon, QALY-weight decrements, AMI and stroke costs, drug treatment costs, and prevalence of hypertension. Substantial variations in the QALY-weight decrements, AMI and stroke costs, and drug treatment costs have only a modest impact on the estimated ICER. Instead, the analyses reveal that the major uncertainty comes from varying the prevalence of (undetected) hypertension in the screened population and the model time horizon. Assuming a higher prevalence in the screened population lowers the ICER (since this factor would improve the health gains from the screening programme and subsequent treatment), and a longer time horizon (30 years vs. 10 years) also improves the cost-effectiveness (lower ICER). The dashed vertical line represents a cost-effectiveness of 500,000 SEK per QALY (€48,500), which is often used as an informal threshold value in Swedish health policy, and as seen, the ICER never fall below that threshold value in any of the sensitivity analyses.

**Probabilistic sensitivity analysis**

Figure 3 shows the cost-effectiveness acceptability curve (CEAC) based on the probabilistic sensitivity analysis. The probability that the screening programme is cost-effective is approximately 2% at a willingness to pay per QALY of 500,000 SEK. At a willingness to pay per QALY of 1 million SEK, the likelihood that the screening programme is cost-effective is approximately 5%.

**Discussion**

This is one of the few studies on the cost-effectiveness of screening for hypertension. The study model was built to capture the costs and outcomes of a programme for opportunistic screening of a general population in a “real-life” scenario, namely, an existing (dental-care) organization; such a setup has been recommended as a potential method of holding screening costs down. Blood pressure sampling was performed in the dental clinic, in the home environment, and in the PHC for at least 10 different days, which reduced the number of false positives by 85% [10]. The screening was performed on a previously unscreened population, which resulted in a large proportion (8%) of newly diagnosed cases being detected. Despite the above-mentioned good conditions, the model results for cost-effectiveness show a very high cost per gained QALY.

**Short-term analysis**

The cost of the screening programme from the perspective of the dental- and health-care payer was 0.6 million SEK (€58,000) and with the addition of socio-economic costs rose to 1.6 million SEK (€154,000). The major cause of the difference in the two sets of costs is the inclusion of the patients’ time cost of BP testing in the societal perspective.

The results of the short-term analysis show that the additional cost was approximately 4,800 SEK (€470) per newly discovered case in the form of dental- and health-care costs and approximately 12,800 SEK (€1,240) per newly discovered case with the inclusion of all societal costs. A similar study of a less effective opportunistic BP screening resulted in an NNS of 18, a PPV of 30%, and a direct cost of 5,300 SEK (€515) per newly discovered case [17].

**Long-term analysis**

The long-term consequences were analysed in a Markov cohort model, with the results showing a cost per QALY of approximately 2.2 million SEK (€210,000). When the patient’s time cost was included to yield the societal perspective, the cost per QALY increased to over 2.8 million SEK (€270,000). This is substantially above the standard threshold values for the cost per QALY referenced in the Swedish health policy literature, where, e.g., the National Board of Health and Welfare defines a cost per QALY below 500,000 SEK as moderate, a cost per QALY in the interval of 500,000 to 1 million SEK as a high cost per QALY, and everything above 1 million SEK per QALY as very high [18]. In the sub-group analyses for men and women, the cost per QALY was lower for men (1.3–1.6 million SEK per QALY) than for women (4.3–5.7 million SEK per QALY). The lower cost per QALY in the cohort of men is primarily explained by the higher prevalence of AMI among men, especially in the relatively younger age groups (and thus a higher potential benefit of screening and drug treatment). However, even in an all-male cohort, the cost per QALY is above the standard threshold levels referenced in the Swedish health policy literature.

The sensitivity analyses show that the prevalence of hypertension and the time horizon have the greatest impact on the model’s results. Sweden has a relatively low prevalence of hypertension (27%) and a well-developed health-care system, which means that many people with hypertension are already identified, which reduces the cost-effectiveness of adding screening. The time horizon in the baseline analysis was 20 years, and extending the horizon to 30 years improved the cost-effectiveness results somewhat (i.e., reduced the cost per QALY), though the cost remained above 1 million SEK per QALY.

The treatment of AMI and stroke has improved over time with improved survival, which also actually (relatively) reduces the value of screening and preventive treatment. Further, most of the 170 newly identified persons had mild hypertension (grade 1), which can partly explain the high to very high cost per QALY from this screening programme. However, it should be noted that the health consequences considered from hypertension were limited to AMI and stroke. Should other consequences such as heart failure, renal failure, atrial fibrillation, cognitive impairment and dementia also be included, it is possible that the cost-effectiveness of the screening programme would be higher.

**Limitation**

The transition probabilities (stroke and AMI risks) were based on risk models from the US Framingham study, and despite being widely used, they may have drawbacks in terms of validity for the given health context in this study [14]. As in all modelling-based studies, some simplifications had to be made that may have had some impact on the results. For example, an individual who has had an AMI can later suffer from stroke or vice versa, which our model did not allow for. An additional limitation with Markov cohort models is that average costs per, e.g., stroke and AMI, are assigned for each case and do not necessarily represent the costs in this particular cohort of patients.

In the data set for our health economic analysis, there are no people with diabetes (as the condition was an exclusion criterion) and no information on cholesterol. The calculations refer to a population without diabetes. The mean values of serum cholesterol for men (4.0) and women (3.2) were used [19].

**Conclusions**

Despite the success of a blood pressure screening programme in identifying a substantial number of true positive hypertension patients in an existing dental-care facility, the cost per QALY was 2.2 million SEK (€210,000), which is considered a high cost. The results thus suggest that adding blood pressure screening in the dental-care setting is not cost-effective.

**Acknowledgements**

The authors would like to thank the participants of the dental health and primary health clinics for their time and effort. They also send warm thoughts to Professor Björn Lindgren for his help with the design of the health economic analysis.

**References**

1. Lim SS, et al. A comparative risk assessment of burden of disease and injury attributable to 67 risk factors and risk factor clusters in 21 regions, 1990-2010: a systematic analysis for the Global Burden of Disease Study 2010. *Lancet.* 2012;380(9859):2224-60.
2. Williams B, Mancia G, Spiering W, et al; Authors/Task Force Members. 2018 ESC/ESH Guidelines for the management of arterial hypertension: the Task Force for the management of arterial hypertension of the European Society of Cardiology and the European Society of Hypertension*. J Hypertens*. 2018;36(10):1953-2041.
3. Gaziano TA, Bitton A, Anand S, Weinstein MC, International Society of Hypertension. The global cost of nonoptimal blood pressure. *J Hypertens* 2009; 27:1472–77.
4. NCD Risk Factor Collaboration. Worldwide trends in blood pressure from 1975 to 2015: a pooled analysis of 1479 population-based measurement studies with 19.1 million participants. *Lancet* 2017;389:37–55.
5. Lindholm LH and Carlberg B. *Moderately elevated blood pressure*. A systematic literature review. Volumes1 & 2. Stockholm: The Swedish Council on Technology Assessment in Health Care 2004. SBU-rapport 170=1:1–514 and 170=2:1–248. 2004.
6. Ettehad D, Emdin CA, Kiran A, Anderson SG, Callender T, Emberson J, et al. Blood pressure lowering for prevention of cardiovascular disease and death: a systematic review and meta-analysis. *Lancet* 2016; 387:957–67.
7. Swedish Social Insurance Agency, Social insurance report. 2012.
8. Engstrom S, Berne C, Gahnberg L, Svardsudd K. Efficacy of screening for high blood pressure in dental health care. *BMC Public Health* 2011; 11: 194.
9. Greenberg BL, Glick M. Assessing systemic disease risk in a dental setting: a public health

perspective. *Dent Clin North Am* 2012 Oct;56(4):863-74.

10. Andersson H, Hedström L, Bergman S and Bergh H.I The outcome of Two-step blood-pressure

screening in dental healthcare. *Scandinavian Journal of Public Health* 2018; 1-7.

11. Brouwer W, Rutten F, Koopmanschap M. *Costing in economic evaluations.* In Drummond M,

McGuire A (eds.) Economic Evaluation in Health Care. Merging Theory with Practice. Oxford: Oxford University Press, 2001:68-93.

12. Ng N, Carlberg B, Weinehall L, Norberg M. Trends of blood pressure levels and management in

Västerbotten County, Sweden, during 1990-2010. *Global Health Action* 2012;5

13. Fidalgo ASQ, Vollenweider P, Marques-Vidal P. Ten-year incidence of hypertension in a Swiss

population-based sample Incidence of hypertension in Switzerland. J Hum Hypertens. 2019; 33(2).

14. Anderson KM, Odell PM, Wilson PWF, Kannel WB. Cardiovascular disease risk profiles. *American Heart Journal* 1991;121:293-98.
15. Tandvårds och läkemedelsförmånsnämndens allmänna råd om ekonomiska utvärderingar, 2015:1”. <https://web.archive.org/web/20170106173601/http://www.tlv.se/Upload/Lagar_och_foreskrifter/TLVAR_2015_1.pdf>
16. Pliskin, Joseph S., Donald S. Shepard, and Milton C. Weinstein. (1980). “Utility Functions for Life Years and Health Status,” Operations Research 28, 206–24.
17. Engstrom S, Borgquist L, Berne C, Gahnberg L, Svardsudd K. Can costs of screening for

hypertension and diabetes in dental care and follow-up in primary health care be predicted? Ups *J Med Sci*. 2013;118:256–62.

18. Socialstyrelsen Nationella riktlinjer för hjärtsjukvård. Hälsoekonomiskt underlag (bilaga). Stockholm, 2015. Artikelnr 2018-6-28
19. Balder JW, de Vries JK, Nolte IM, Lansberg PJ, Kuivenhoven JA, Kamphuisen PW. Lipid and

lipoprotein reference values from 133,450 Dutch Lifelines participants: Age- and gender-specific baseline lipid values and percentiles. *Journal of Clinical Lipidology* 2017; doi.org/10.1016/j.jacl.2017.05.007.

20. SCB Available from: <https://www.scb.se/hitta-statistik/statistik-efter-amne/befolkning/befolkningens->sammansattning/befolkningsstatistik
21. Eriksson M, Carlberg B, Eliasson M. The disparity in long-term survival after a first strike in patients with and without diabetes persists: the Northern Sweden MONICA study. *Cerebrovascular Diseases* 2012;34:153-60.
22. Isaksson RM, Jansson JH, Lundblad D, Näslund U, Zingmark K, Eliasson M. Better long-term

survival in young and middle-aged women than in men after a first myocardial infarction between 1985 and 2006, an analysis of 8630 patients in the Northern Sweden MONUCA study*. BMC Cardiovascular Disorders* 2011;11:1.

23. Andersson H, Berg H, Lindgren B. Slutrapport- Blodtrycksscreening i tandvården. Halmstad: Region Halland 2017.
24. Lanitis T, Kongnakorn T, Jacobson L, De Geer A. Cost-effectiveness of apixaban versus warfarin and aspirin in Sweden for stroke prevention in patients with atrial fibrillation. *Thrombosis Research* 2014;134:278-87.
25. Tengs TO, Yu M, Luistro E. Health-related quality of life after stroke. A comprehensive review.

*Stroke 2001*;32:964-972.

26. Post PN, Stiggelbout AM, Wakker PP. The utility of health states after stroke. A systematic review of the literature. *Stroke 2001*;32:1425-29.
