# Supplementary material for: The cost-effectiveness of a two-step blood pressure screening programme in a dental health-care setting
Source: PLoS One. 2021 May 25;16(5):e0252037. doi: 10.1371/journal.pone.0252037 (PMC8148372; doi:10.1371/journal.pone.0252037)
Supplement: Attachment — Submitted filename: Response to Reviewers 210214.docx [file pone.0252037.s003.docx]

**Comments regarding Manuscript PLOS ONE Decision: Revision required [PONE-D-20-30890] - [EMID:7424c05fb27b3082] entitled ” The cost-effectiveness of a two-step blood pressure screening programme in a dental health-care setting".**

Thank you for your most valuable comments on our manuscript. We found the comments useful and have made a revision of the paper. The proposed changes are marked in the manuscript and I have also specifically addressed each of the points made by the reviewers in the areas provided. I have uploaded my manuscript in a clean copy, the changed figures and tables. I have also uploaded a revised manuscripts, with markings that describe the changes I have made. Hopefully you are satisfied with our changes of the manuscript, tables and figures otherwise please contact us again so we can do further changes.

Best regards

Helen Andersson

I have address these requirements and I hope a have understood them right.

1. Please ensure that your manuscript meets PLOS ONE's style requirements, including those for file naming. The PLOS ONE style templates can be found at

<https://journals.plos.org/plosone/s/file?id=wjVg/PLOSOne_formatting_sample_main_body.pdf> and

<https://journals.plos.org/plosone/s/file?id=ba62/PLOSOne_formatting_sample_title_authors_affiliations.pdf>

 2. Please provide additional details regarding participant consent. In the Methods section, please ensure that you have specified (1) whether consent was informed and (2) what type you obtained (for instance, written or verbal). If your study included minors, state whether you obtained consent from parents or guardians. If the need for consent was waived by the ethics committee, please include this information.

Comment: Thanks, We have clarified participants consent in line 97,98. Both written and oral consent was obtained and the study is approved by the ethical review board in Lund, No. 2013/553 and 2015/446.

3. PLOS requires an ORCID iD for the corresponding author in Editorial Manager on papers submitted after December 6th, 2016. Please ensure that you have an ORCID iD and that it is validated in Editorial Manager. To do this, go to ‘Update my Information’ (in the upper left-hand corner of the main menu), and click on the Fetch/Validate link next to the ORCID field. This will take you to the ORCID site and allow you to create a new iD or authenticate a pre-existing iD in Editorial Manager. Please see the following video for instructions on linking an ORCID iD to your Editorial Manager account: <https://www.youtube.com/watch?v=_xcclfuvtxQ>

 4. Please include a separate caption for each figure in your manuscript.

Comment: I have included separate caption for each figures.

5. Please include your tables as part of your main manuscript and remove the individual files. Please note that supplementary tables (should remain/ be uploaded) as separate "supporting information" files

Comment: I have include my tables in my main manuscript and removed the individual’s files.

**Reviewer #1**: Authors developed an economic model to assess the cost-effectiveness of an opportunistic hypertension screening programme compared to no screening of people aged 40-75 attending a dental-care facility. The authors found that under the current model structure and assumptions that opportunistic screening for hypertension in a dental care setting in a country with a relatively low prevalence is unlikely to be cost-effective.

In general, the manuscript was well written and structured. The abstract provides an accurate summary of the cost-effectiveness analysis. The conduct of the economic analysis conformed to best practices, but there some queries/suggestions.

• Given that the economic analysis considers the 40-74 years old population. Would it be helpful to include in the introduction the prevalence of hypertension in this population in Sweden?

Comment: Thanks, We have clarified the prevalence in the introduction (lineThank you for your comments.

Tack för dina kommentarer. Vi har gjort ändringar

Thank you for your synpnkter.

Tack för att synpnkterVi gjorde ändringar

Det gick inte att läsa in hela resultatet

Försök igen

Försöker igen …

Försöker igen …

We have made changes

Vi har gjort ändringar

Det gick inte att läsa in hela resultatet

Försök igen

Försöker igen …

Försöker igen …

54, 55): the prevalence increases with age from 12% in young adults to 56% in the elderly [5].

• The intervention includes blood pressure readings taken by a dental health nurse, with some requiring home blood pressure measurements. Please can the authors elaborate on if participants were supplied with blood pressure monitors?

Comment: Thanks, we have clarified the home blood pressure measurement, lineThank you for your comments.

Tack för dina kommentarer. Vi har gjort ändringar

Thank you for your synpnkter.

Tack för att synpnkterVi gjorde ändringar

Det gick inte att läsa in hela resultatet

Försök igen

Försöker igen …

Försöker igen …

We have made changes

Vi har gjort ändringar

Det gick inte att läsa in hela resultatet

Försök igen

Försöker igen …

Försöker igen …

88, 89

• Please can the authors provide the reference used to convert Swedish Kronor to Euros? It appears that some costs were obtained from the literature, please can the authors reference how these were inflated to current prices?

Comment: We have now added a reference to the conversion (end of cost-effectiveness section) and also highlight that we used the consumer price index to translate all costs into 2019 prices.

• Please can the authors state in the methods the software program that was used to undertake the economic analysis?

Comment: The model was built and analyzed in Microsoft Excel and Stata, which is now specified in the end of the section called “Cost-effectiveness”.

• The queries here relate to Table 1. First, the authors have included a cost of 165,000 SEK for vaccine program administration. Please can the authors elaborate on this cost included? Second, the authors state ‘formal care costs: short-term model.’ However, in my copy of the manuscript I have not seen this ‘short-term model’. Apologies if I have missed it. Third, it was not clear what was the average age of the population. Fourth, what utility value is used for people in the healthy state? Please can the authors expand on the abbreviations used in table 1?

Comment: A clarification about the cost is made in table 1. Mean age is supplemented in line 93. We apalogize for the confusion with the “short-term model” terminology, which is what we refer to as the “short-term analysis” in the manuscript. We have now changed the terminology in table 1 to match what we use in the manuscript. We set the utility value for people in the health state to 1, but only report the utility decrements since we are only presenting qaly outcomes in differences between the alternatives (i.e. do not really “care” about the total number of qalys generated in the two treatment alternatives, only the difference).

• Based on the illustrative Markov model structure, it appears that people can only experience one event (e.g. cannot experience more than one stroke). Should this assumption be highlighted?

Comment: Yes, this is an assumption we make and we agree that it should be spelled out. We have now included a statement about this in the paragraph describing the “long-term analysis”.

• Figure 1 presents the results of the one-way sensitivity analysis in the form of a tornado diagram. Please can the authors include the legend?
Comment: Sorry if the legends not was included. We have secured the legends in all the figures.

• I presume that with the two-step screening program, this will lead to early detection of people with hypertension compared to usual practice. Hence, it would be useful to show the impact of early detection, by reporting the number of events (e.g. stroke and AMI) that would be saved if this two-step program was implemented in practice.

Comment: The model results show that the two-step screening program (over the 20 years) would reduce the number of AMIs by 1.5 and the number of strokes by 0.7. This is now added as a note to the results in Table 3.

• The queries here relate to the probabilistic sensitivity analysis (PSA). First, it was not clear what uncertainty range and distribution were used around the prevalence. Second, if space allows, please can the authors report the PSA results in the form of a scatterplot? Third, please consider re-phrasing the PSA results to, ‘The probability that the screening programme is cost-effective is approximately 0.02 at a willingness-to-pay threshold of 500,000 SEK per QALY.’

Comment: We have now introduced a scatterplot/cost-effectiveness plane to represent the results from the PSA as well. We have also adjusted the text in accordance to the suggestion by the reviewer. The range for the distribution of the prevalence is now listed in the last para of “Assessing uncertainty”.

**Reviewer #2:** This is a well conducted study that is relevant in its field. Requirements for an economic evaluation according to the Drummond checklist were met.
The results could be presented in a less confusing way by indicating the ICER in both currencies for each perspective sequentially as opposed to putting them in brackets (page 1 line 32-35, page 6 line 200-204).

Comment: Thanks for your advice. We have made clarifications about ICER for each perspective, lineThank you for your comments.

Tack för dina kommentarer. Vi har gjort ändringar

Thank you for your synpnkter.

Tack för att synpnkterVi gjorde ändringar

Det gick inte att läsa in hela resultatet

Försök igen

Försöker igen …

Försöker igen …

We have made changes

Vi har gjort ändringar

Det gick inte att läsa in hela resultatet

Försök igen

Försöker igen …

Försöker igen …

33-36 and 216-221

- Also indicate the currency year used for the Euro conversion.

Comment: This is now included in the last para of the section on “Cost-effectiveness analysis”.

- Mention the time horizon in specific terms as opposed to simply indicating short-term and long term especially at first mention and in the abstract (page 1 line 24-25, page 3 line 95, 112)

Comment: Thanks, We have made clarifications about the short-term and the long term both in abstract and in the method (lineThank you for your comments.

Tack för dina kommentarer. Vi har gjort ändringar

Thank you for your synpnkter.

Tack för att synpnkterVi gjorde ändringar

Det gick inte att läsa in hela resultatet

Försök igen

Försöker igen …

Försöker igen …

We have made changes

Vi har gjort ändringar

Det gick inte att läsa in hela resultatet

Försök igen

Försöker igen …

Försöker igen …

23-26 and 100-102): the short-term (from screening until diagnosis has been established) and long-term (20 years)

- Provide references for the model assumptions, especially those that are specific to the Swedish population (e.g page 4 line 130-133)

Comment: Has changed in table 1. Vaccin program administration, an unfortunate translation, it should be a screening program administration.

- Minor comment: Regarding the Markov model, dead is an absorbing state so you do not expect a repeat arrow

Comment: Thanks, this is now fixed.

**Reviewer #3**: This is a well written manuscript assessing the cost-effectiveness of a screening programme for hypertension in a dental health care setting compared to the "status quo". While I find the short term analysis easier to follow, further detail is required on the long term modelling approach so that I can assess the validity of the methods and the subsequent results. I provide some more specific comments below:

*Short term analysis:*
- Would be helpful to provide some more details on any potential false positives in the screening programme arm (or are there assumed to be none?)

Comment: We have clarified the part about false positive in the screening programme arm (lineThank you for your comments.

Tack för dina kommentarer. Vi har gjort ändringar

Thank you for your synpnkter.

Tack för att synpnkterVi gjorde ändringar

Det gick inte att läsa in hela resultatet

Försök igen

Försöker igen …

Försöker igen …

We have made changes

Vi har gjort ändringar

Det gick inte att läsa in hela resultatet

Försök igen

Försöker igen …

Försöker igen …

140-146): “Since the diagnosis of hypertension was based on repeated blood pressure measurements both at home and in clinic (screening arm), it was assumed that no one in the screening arm was false positive.

Among those diagnosed with high blood pressure based on blood pressure screening in a clinical setting (comparing arm), we estimate that approximately 15 individuals (25%) present false positives (that is, they display white coat hypertension [WCHT]), and the remaining 46 (75%) are expected to be true positives [17].”

- Data on the differential identificationis based on assumptions of the control arm. Please provide more details of the data used to inform these assumptions.

Comment: Thanks, We have clarified these assumptions (line 143) with the following text: “Among those diagnosed with high blood pressure based on blood pressure screening in a clinical setting (comparing arm), we estimate that approximately 15 individuals (25%) present false positives (that is, they display white coat hypertension [WCHT]), and the remaining 46 (75%) are expected to be true positives [17]”.

Tack för dina kommentarer. Vi har gjort ändringar

Thank you for your synpnkter.

Tack för att synpnkterVi gjorde ändringar

Det gick inte att läsa in hela resultatet

Försök igen

Försöker igen …

Försöker igen …

We have made changes

Vi har gjort ändringar

Det gick inte att läsa in hela resultatet

Försök igen

Försöker igen …

Försöker igen …

- It would be helpful to comment on the incremental analysis- it is not really screening programme or status quo, it is screening programme in addition to the status quo. How would this affect your analysis?

Comment: It is correct that it is “screening program additional to status quo” vs “status quo”. It would not change our analyses, but we have rephrased the terminology (in abstract as well as in the Methods section) to clarify the evaluation question in line with this.

- What is the time horizon of the short term analysis?

Thanks, We have made clarifications about the short-term and the long term both in abstract and in the method (lineThank you for your comments.

Tack för dina kommentarer. Vi har gjort ändringar

Thank you for your synpnkter.

Tack för att synpnkterVi gjorde ändringar

Det gick inte att läsa in hela resultatet

Försök igen

Försöker igen …

Försöker igen …

We have made changes

Vi har gjort ändringar

Det gick inte att läsa in hela resultatet

Försök igen

Försöker igen …

Försöker igen …

23-26 and 100-102): the short-term (from screening until diagnosis has been established) and long-term (20 years).

How many of the patients not identified in the control arm in this period would go on to be identified over the longer term by routine practice? What impact would this have on your results?
Comment: We have described this assumption in line 140-143, and 316-318: “And we have assumed that there is no difference between the two treatment alternatives in long-term identification of additional hypertension patients”..

*Long term analysis:*
- Are there separate Markov models for those with identified hypertension, non identified hypertension and no hypertension or are you taking an average? The use of a markov model approach suggests a homogenous population entering the model at the beginning, as such if may not be a suitable approach if you do not model these 3 heterogeneous groups separately.

Comment: Everyone enters in the “healthy state” but with different stroke or AMI risks depending on their hypertension status, so in that sense our model is a (weighted) average from different sub-populations. We agree that we could have constructed separate Markov models for the three sub-categories (in each arm), run them separately, and in the end weighted them together to get expected costs and QALYs in the screening arm vs the status quo arm. However, in this context this gives us identical results with regards to the expected costs and QALYs in the two alternative arms that we compare.

- Can unidentified patients in the model become identified?

Comment: If referring to unidentified with hypertension becoming identified with hypertension during the time frame of the model, the answer is no, which is a simplifying assumption in our model. Adding this to our model would change the situation in both arms, and we have now added this as a limitation in the discussion section.
