# Supplementary material for: The cost-effectiveness of a two-step blood pressure screening programme in a dental health-care setting
Source: PLoS One. 2021 May 25;16(5):e0252037. doi: 10.1371/journal.pone.0252037 (PMC8148372; doi:10.1371/journal.pone.0252037)
Supplement: Attachment — Submitted filename: Response to Reviewers 210505.docx [file pone.0252037.s004.docx]

**Comments regarding Manuscript PLOS ONE Decision: Revision required [PONE-D-20-30890R1] - [EMID:5dbfc31d1d9edccb] entitled ” The cost-effectiveness of a two-step blood pressure screening programme in a dental health-care setting".**

Thank you for your most valuable comments on our manuscript. We found the comments useful and have made a revision of the paper. The proposed changes are marked in the manuscript and I have also specifically addressed each of the points in the areas provided. I have uploaded my manuscript in a clean copy, the changed figures and tables. I have also uploaded a revised manuscripts, with markings that describe the changes I have made. Hopefully you are satisfied with our changes of the manuscript, tables and figures otherwise please contact us again so we can do further changes.

Best regards

Helen Andersson

**Response to Reviewers**

Page 3 line 18-21: This belongs to the results section.

Comments: Thanks, we agree and have moved this sentence to the results section. Page 6 line 199-202

Page 3 line 25-29: Sentence is very long, Split sentence into two. Indicate the actual time for short-term in years or months.

Comments: Thanks, We have split the sentence, page 3 line 97-101 and we have clarified the actual time for short-term in months, page 3 line 99.

Page 3 line 30: The abbreviation is preceded by the word in full.

Comments: Thanks, We have made this change in line 103.

Page 4 line 2-5: Indicate the source of costing data.

Comments: We have clarified by indicate the source of costing data, ref 11, page 3 line 114.

Page 4 line 11-16: Indicate source of costing data and how non-health care costs were obtained.

Comments: We have clarified by indicate the source of costing data, ref 15, page 4 line 125.

Page 4 line 38: write AMI in full at first time of mention.

Comments: I have write AMI in full at fist time of mention in page 3 line 110.

Page 5 line 9-11: Indicate how QALYs were obtained, was an instrument used, and frequency of QoL assessment

Comments: We have clarified this in page 5 line 159- 166. “In this model based study the health outcomes are measured in terms of quality-adjusted life years (QALYs), which combine health-related quality of life (QALY weights) and life length. QALY-weight decrements, is based on published evidence, associated with a stroke and AMI event”.

Page 5 line 29-30: Indicate the threshold value for Sweden.

Comments: We have clarified and indicate the threshold value for Sweden, page 5 line 181-183.

Results

Page 6 line 5: First time the link between this previous study and this present study is mentioned. This needs to be clearly spelt out in the introduction that this CEA is a follow-on from that previous study.

Comments: Thanks, we have clarified the link between the previous study and this present study in Introduction, page 2 line 75-76. “We have used the results from the opportunistic two step screening of hypertension to conduct this follow-up cost effectiveness analysis”...

Table 2: Include a column for unit costs.

Comments: The unit cost for all cost items are listed in table 1. We have clarified this with a note in table 2.
